# UVB Irradiation as a Human Pain Model—A Scoping Review

**DOI:** 10.3390/life16040662

**Published:** 2026-04-13

**Authors:** Almuth Lang, Sascha Hammer, Thomas Danninger, Johanna Lang, Beate Averbeck, Shahnaz Christina Azad, Helmar Bornemann-Cimenti

**Affiliations:** 1 Institute of Cardiovascular Physiology and Pathophysiology, Ludwig Maximilian University, 80539 Munich, Germany; almuth.lang@campus.lmu.de (A.L.); beate.averbeck@med.uni-muenchen.de (B.A.); 2 Department of Anesthesiology and Intensive Care Medicine, Medical University of Graz, 8010 Graz, Austria; sascha.hammer@medunigraz.at; 3 Department of Anesthesiology, Perioperative Medicine and General Intensive Care Medicine, Paracelsus Medical University, 5020 Salzburg, Austria; 4 Interdisciplinary Pain Unit, University Hospital LMU Munich, 81377 Munich, Germany; shahnaz.azad@med.uni-muenchen.de

**Keywords:** pain model, UV-B, sunburn, hyperalgesia, sensitization

## Abstract

The ultraviolet B (UVB) sunburn model is a well-established human experimental pain paradigm for investigating underlying inflammatory pain mechanisms and is used in preclinical drug development research. This scoping review aimed to provide an overview of how UVB-induced cutaneous inflammation has been applied across experimental studies, with particular emphasis on methodological characteristics, sensory outcomes, and reported safety aspects. A total of 12 studies published between 1999 and 2025, comprising 367 participants, met the inclusion criteria. Across all studies, UVB irradiation produced a clearly demarcated inflammatory response accompanied by pronounced primary hyperalgesia. Peak primary hyperalgesia was typically observed between 24 and 48 h following irradiation and remained detectable for at least 72 h. Heterogeneity was identified using UVB dose calibration strategies, spectral properties of the irradiation source, size and anatomical location of the irradiated area, timing of sensory assessment, and applied testing methodologies. In contrast to the consistent induction of primary hyperalgesia, secondary hyperalgesia was reported inconsistently and appeared to depend on methodological conditions. Post-inflammatory hyperpigmentation was reported primarily after irradiation with three minimal erythema doses, whereas lower doses appeared to provide a more favorable balance between hyperalgesia induction and tolerability. Overall, the UVB sunburn model reliably induces primary inflammatory hyperalgesia in humans. However, careful selection and standardization of methodological parameters are essential to optimize its use in mechanistic and early-phase analgesic research.

## 1. Introduction

Experimental human pain models are essential for investigating the underlying mechanisms of pain. Furthermore, the models are used in the initial phase of clinical drug development to assess analgesic effects and determine effective dose ranges [1,2]. Among the various experimental models available, the sunburn pain model using ultraviolet B (UVB) irradiation is well established. It reliably induces a well-defined and reproducible cutaneous inflammatory reaction. This response is characterized by primary hyperalgesia, including reduced mechanical and thermal pain thresholds and exaggerated responses to suprathreshold stimuli, without causing tissue destruction or significant spontaneous pain [3,4,5,6,7,8,9]. UVB irradiation (290–320 nm), the most erythemogenic spectral component of sunlight [4], is predominantly absorbed within the epidermis [7]. It produces a sharply demarcated erythematous lesion with localized hypersensitivity, reflecting the sensitization of peripheral nociceptor terminals [8,10,11]. Although secondary hyperalgesia has been reported in several studies, its detectability remains inconsistent [12], highlighting the need for clearer methodological standardization when the model is used to investigate central sensitization. Beyond the UVB model itself, several other experimental pain models—such as the capsaicin pain model, the heat/capsaicin sensitization model, or hypertonic saline injections—are commonly used in human pain research. While both UVB and capsaicin induce robust heat pain sensitization, their broader quantitative sensory testing (QST) response profiles differ, indicating that these models are not interchangeable alternatives [13].

Methodological heterogeneity presents major challenges for interpreting and comparing findings across UVB sunburn studies. Variability arises from differences in dose calibration (fixed physical dose versus individual minimal erythema dose), spectral range of the light source, anatomical location and size of the exposed skin, quantitative sensory testing methods, assessment frequency, and duration of follow-up assessment. These factors contribute to inconsistent reports regarding time courses and the intensity of erythema, perfusion, and sensory changes. Safety aspects have received comparatively little attention, even though long-lasting post-inflammatory hyperpigmentation has been observed after high-dose UVB exposure [2].

Against this background, a structured synthesis is needed to clarify current experimental approaches, identify methodological sources of variability, and highlight knowledge gaps that constrain model standardization. Accordingly, this scoping review aims to map how UVB-induced sunburn pain models have been applied in human research, with a focus on dose paradigms and safety considerations; spectral characteristics; temporal profiles of inflammatory and sensory changes; and conditions influencing the expression of primary and secondary hyperalgesia. This synthesis seeks to facilitate greater methodological consistency and to provide a clearer foundation for future research employing the UVB sunburn model in the study of inflammatory pain and sensitization, as well as in pharmacological studies.

## 2. Materials and Methods

This scoping review was designed and conducted following the methodological recommendations of the PRISMA extension for scoping reviews (PRISMA-ScR) and the Joanna Briggs Institute guidelines for evidence synthesis [14,15]. A review protocol was registered at the Open Science Framework (DOI: 10.17605/OSF.IO/Y5PW3) before conducting the scoping review. The PRISMA checklist is available as Supplement Table S1. Because the review relies exclusively on previously published studies and does not involve primary data collection, formal ethical approval was not required.

### 2.1. Research Question

The aim of this scoping review was to synthesize and describe how the sunburn pain model has been applied in human experimental research. A scoping review design was chosen because, although the sunburn pain model has been well established, its application has varied widely across studies. These differences include spectral composition, dosing strategies, size and site of irradiation, measurement time points, observation periods, and reported outcomes.

### 2.2. Search Strategy

A comprehensive literature search was performed using PubMed, Ovid (MEDLINE, Cochrane Database and EMBASE), and CINAHL. All databases were searched from their respective inception dates to the final search on 16 October 2025 The complete search strategies for each database are provided in Table 1.

Additionally, the literature was searched in Google Scholar with the keywords “sunburn pain model hyperalgesia”.

### 2.3. Study Selection

All records identified through the database searches were imported into the Rayyan web application (www.rayyan.ai, accessed on 9 April 2026) and automatically checked for duplicates. Two independent reviewers (AL and SH) screened the titles and abstracts using Rayyan, and in cases of disagreement, the record was retained for the next screening stage. The full texts were then assessed independently by the same reviewers, and any conflicts were resolved through discussion with a third reviewer. To ensure completeness, forward citation tracking using Google Scholar was performed to identify additional relevant studies.

Eligibility criteria were defined as a priori. Human studies of any age group were included, whereas studies involving animals, cellular systems, or other in vitro models were excluded. Interventions had to include a protocol using UVB irradiation (any dose or wavelength range) with the intention to induce pain. Studies applying ultraviolet radiation for therapeutic purposes were excluded. Comparator conditions such as pre- vs. post-intervention measurements; within-subject comparison using unexposed skin (e.g., contralateral sites); or separate control groups were eligible. No outcome-based exclusion criteria were defined. Eligible study designs comprised prospective experimental studies, including randomized controlled trials, crossover studies, split-site or split-body designs, and other comparable human experimental designs. Reviews, meta-analysis, conference abstracts, proceedings, letters without original data, and editorials were excluded.

### 2.4. Data Extraction

Data extraction was performed by one reviewer using a structured Excel spreadsheet (Excel 365, Microsoft Cooperation, Redmond, WA, USA), and the extracted data were independently checked by a second reviewer. Extracted data were synthesized narratively and summarized in a table and grouped by authors; year of publication; country of correspondence; study design; sample size; details of the UVB sunburn pain model (wavelength, UV dose, localization, and size of the irradiated area); measurement time points; and study aims.

### 2.5. Risk of Bias Assessment

Risk of bias was evaluated with the ROBIN-I assessment tool. This approach was considered appropriate because the included studies predominantly used non-randomized experimental designs, including crossover and within-subject designs, for which tools developed for parallel-group randomized trials are not suitable. When applying ROBIN-I, we paid particular attention to design-specific aspects, including possible carryover effects, the timing of assessments, the use of control sites, and blinding of outcome evaluation. The assessments were performed by one reviewer and independently checked by a second reviewer.

## 3. Results

**Figure 1:**
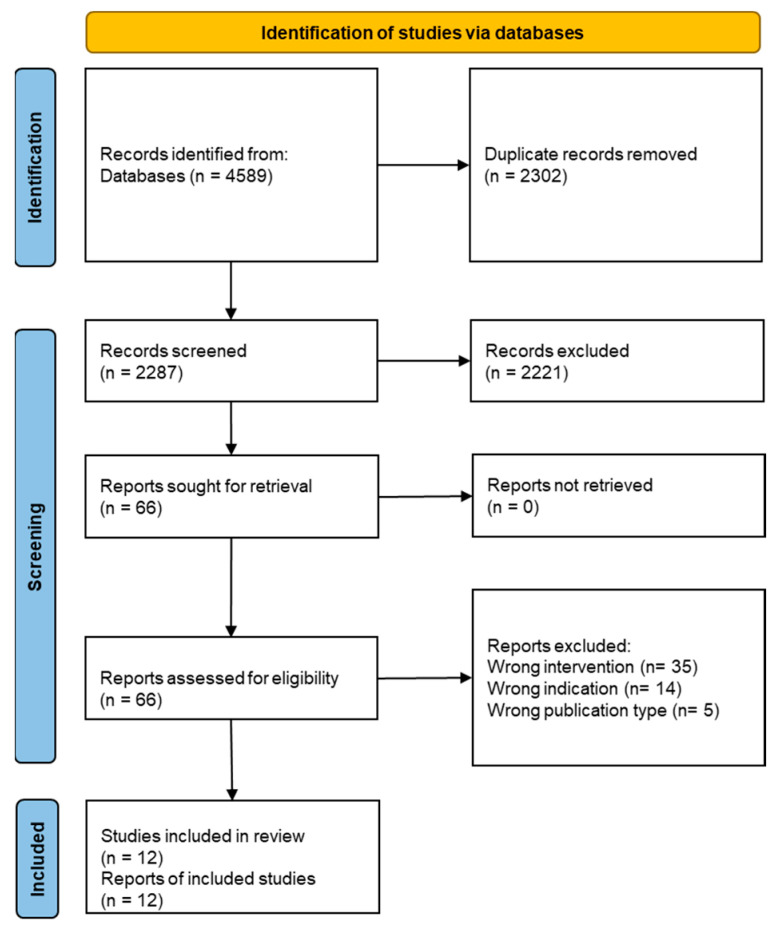
Flowchart of the study.

A total of 4589 records were identified across all databases. After the removal of duplicates, the remaining 2287 records were screened by their titles and abstracts, yielding 66 publications for full-text assessments. Following full-text screening and resolution of disagreements by discussion with a third reviewer, 12 studies met the inclusion criteria and were included in the final synthesis. Ten studies investigated UVB-induced irradiation, whereas two studies incorporated other spectral conditions, including UVA and solar-simulated radiation (SSR). These are reported for contextual comparison, while the core synthesis focuses on UVB-induced irradiation. Figure 1 presents the flowchart of the study.

The included studies were published between 1999 and 2025 and originated from Germany (*n* = 3) [4,7,13]; Denmark (*n* = 3) [6,12,16]; the United Kingdom (*n* = 2) [5,9]; Austria (*n* = 2) [8,10]; the Netherlands (*n* = 1) [2]; and Australia (*n* = 1) [11]. Across all studies, 367 human participants were included, with individual sample sizes ranging from 8 to 142 subjects. Table 2 presents the studies’ characteristics.

**Figure 2:**
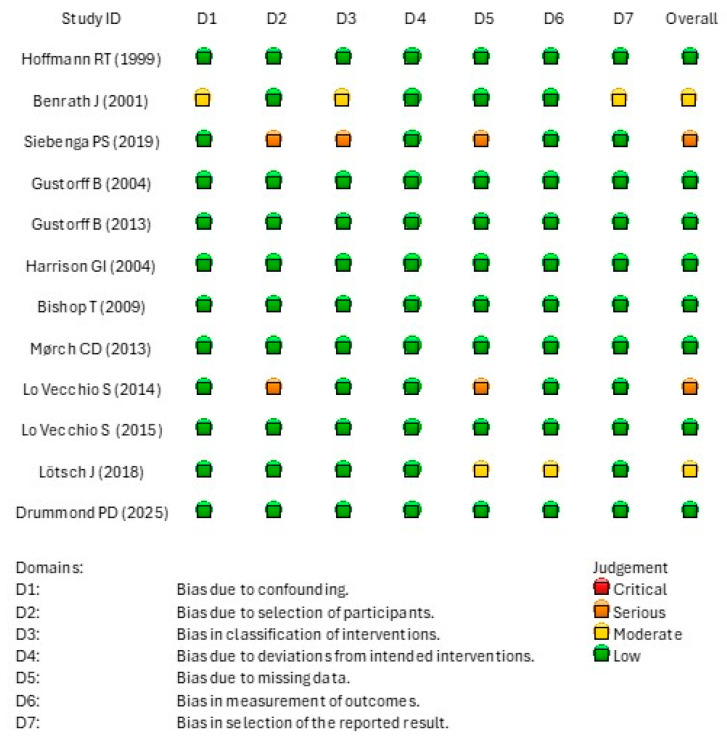
Risk of bias assessment using the ROBINS-I assessment tool.

Risk of bias assessment using the ROBINS-I assessment tool indicated that two studies were rated as having an overall serious risk of bias (Figure 2) [2,6].

## 4. Discussion

The primary focus of this scoping review is the UVB pain model. The studies by Hoffmann et al. and Harrison et al., which also applied UVA and solar-simulated radiation (SSR), were included as comparator conditions to provide context for interpreting UVB-specific findings and did not form the core synthesis [5,7].

The available evidence collectively demonstrates that UVB irradiation induces a well-circumscribed inflammatory reaction accompanied by pronounced primary hyperalgesia, making the sunburn pain model—using ultraviolet B irradiation—a human paradigm for studying inflammatory pain mechanisms under controlled conditions.

However, the magnitude, temporal development, and spatial characteristics of these responses varied across studies. A large part of this variability likely stemmed from methodological differences, such as how the UVR dose was calibrated, what spectral range the light source covered, how often and how long assessments were performed, as well as the size and anatomical location of the irradiated area. To understand and interpret these differences, this article synthesizes the reviewed literature across four key domains: dose paradigms and safety considerations; spectral characteristics of UVR; temporal profiles of inflammatory and sensory changes; and differential expression of primary versus secondary hyperalgesia. Taken together, these factors underline both the reliability of the model and the critical need for standardization when comparing results between studies, or for optimizing protocols for future research.

### 4.1. Dose Paradigms and Safety

The reviewed literature employs two principal dosing strategies that are crucial for interpreting dose–response findings. Some studies used fixed physical doses, giving all participants the same amount of UVB [4,11] or UVA energy [7], whereas others individualized exposure using the minimal erythema dose (MED), defined as the dose that produces a clearly bordered erythema 24 h after irradiation [2,5,6,7,8,9,10,12,13,16]. This distinction is important insofar as MED-based dosing adapts UVR exposure with individual sensitivity and can therefore make dose–response relationships more comparable across participants.

Across the examined studies in this review, erythema-producing doses of UVB irradiation, even 1 MED, already results in increased skin blood flow and decreased mechanical and thermal pain thresholds within the irradiated area, indicative of primary hyperalgesia [4,5,7,9]. Furthermore, higher doses of UVB irradiation result in increased vascular and sensory responses in a dose-dependent manner, i.e., the larger the erythema, the greater the perfusion, and with a greater reduction in pain thresholds [4,7].

Importantly, only one of the reviewed studies addressed safety considerations, particularly with respect to the risk of post-inflammatory hyperpigmentation (PIH) associated with high-dose UVB irradiation. Specifically, while 3 MED results in strong and stable hyperalgesia, it also carries a significant risk of long-lasting post-inflammatory hyperpigmentation [2]. In contrast, 2 MED appears to offer a better balance between inducing stable hyperalgesia and minimizing the risk of PIH [2]. However, the sole study on which this information about UVB-related safety risks—including post-inflammatory hyperpigmentation—is based was found to have an overall serious risk of bias; therefore, these findings should be interpreted with caution.

### 4.2. Spectral Characteristics

Beyond dosing considerations, the differences in spectral composition further contribute to variability in hyperalgesic outcomes. UVB irradiation consistently evokes delayed hyperemia and hyperalgesia, whereas high absolute doses of UVA that are not adjusted for erythemal efficacy fail to produce comparable inflammation and sensory changes [7]. In contrast, Harrison et al. showed that when UVA or SSR (solar-simulated radiation), in which UVB is the most erythemogenic component, are administered at erythema-matched doses, they can induce significant erythema and reductions in mechanical and thermal pain thresholds with only minor differences between the two spectra [5]. These findings indicate that the critical determinant is not the nominal wavelength band alone. Far more important is the degree of erythema, regardless of whether it is induced by UVA or SSR [5].

### 4.3. Temporal Profile of Inflammation and Hyperalgesia

The temporal evolution of erythema, superficial blood flow, and sensory changes across the included studies consistently demonstrates a delayed and highly stable inflammatory response after UVB irradiation.

There is broad agreement that erythema typically develops progressively over the first post-irradiation hours, becoming clearly visible and well-demarcated by approximately 24 h [4,5,6,7,8,9,12], and remaining stable and reliably quantifiable for up to 72 h when assessed by using erythema index measures [16]. These findings indicate that a sharply bordered erythematous response develops up to 24 h and reaches a reliably measurable plateau between 24 and 72 h.

The vascular response shows a similar delayed onset [7] but with more variability in timing and shape. Most MED-based protocols report a clear maximum of skin blood flow around 24 h, as demonstrated by Bishop using 1–3 MED, by Mørch and Lo Vecchio using 3 MED, and by Hoffmann after 1 MED [6,7,9,12,16]. In contrast, other studies have reported an earlier or biphasic response. Increasing the dose from 1 MED to 3 MED in Hoffmann’s study shifted the hyperemic maximum to a time point at 12 h [7]. Furthermore, Benrath et al. observed a biphasic pattern with peaks at 12 and 36 h for all three fixed-dose conditions (133–400 mJ/cm^2^), and showed that the increased blood flow persisted for four days and reached control values only around 216 h post-UVB [4]. Benrath et al. also reported increased vascular responses beyond the irradiated site, a so-called flare reaction [4]. Bishop et al. and Gustorff et al. similarly reported no evidence of an axon-reflex mediated flare and no significant increase in skin blood flow in the surrounding area [8,9]. Overall, 24 h represents the most reproducible peak across studies, and the evidence across the included studies suggests that the timing and shape of the hyperemic response may be modulated by the UVB dose and the frequency of measurement sampling.

Mechanical and thermal hyperalgesia, assessed using heat pain thresholds, also developed with a delay [7], reaching minimal thermal and mechanical thresholds—marking maximal hyperalgesia—between 24 and 48 h post-irradiation [4,5,7,8,16]. Notably, Bishop et al. observed a pronounced peak of hyperalgesia at 24 h, and Lo Vecchio and colleagues also confirmed significant decreases in pinprick and pressure pain thresholds at the same time point [6,9,12]. Results from a multidimensional QST study using machine learning techniques to analyze data demonstrated that the heat pain thresholds represented the most strongly UVB-affected QST parameters [13].

Extending beyond commonly assessed sensory modalities, Gustorff et al., using a broader approach of quantitative sensory testing, additionally found that UVB-induced primary hyperalgesia also encompassed blunt pressure pain, while cold stimulation revealed both cold hyperesthesia and cold pain hyperalgesia within the irradiated skin [8]. Furthermore, Loetsch et al., employing multidimensional quantitative sensory testing, identified UVB-related alterations in cold pain sensitivity [13].

Hyperalgesia remains reliably measurable for at least 72 h [5,16], persisting up to 96 h and returning to control values only after approximately 216 h post-irradiation [4]. Reproducibility studies have demonstrated high within- and between-session stability of both neurogenic inflammation and primary hyperalgesia [16], as well as of secondary hyperalgesia [10]. This temporal stability indicates a robust testing window and is therefore suitable for investigating inflammatory hyperalgesia and evaluating analgesic interventions under controlled conditions.

Across the studies, the temporal patterns of UVB-induced inflammation and hyperalgesia were largely robust and reproducible; however, methodological factors appear to have shaped the precise timing and persistence of these responses. Discrepancies in the reported time course of perfusion and hyperalgesia are largely explained by a limited time resolution and differences in assessment frequency and observation windows. Furthermore, the UVB dose appeared to have an influence on the timing of perfusion response. Taken together, the reviewed literature shows a convergent temporal pattern: hyperemia and hyperalgesia develop with a delay. Erythema reaches a stable plateau between 24 and 72 h; skin blood flow usually peaks around 24 h but can vary when different doses or sampling frequencies are used; and maximal primary hyperalgesia occurs between 24 and 48 h and remains reliably detectable for up to 72 h. This convergence suggests that 24 to 72 h after UVB irradiation is the optimal time window for the standardized assessment of UVB-induced skin inflammation and primary hyperalgesia.

### 4.4. Primary and Secondary Hyperalgesia

In addition, the assessment of primary and secondary hyperalgesia provides insights into the influence of the sunburn pain model on peripheral and central processing mechanisms. The UVB model reliably captures primary hyperalgesia within the irradiated area, which is generally attributed to the sensitization of peripheral nociceptors and local inflammatory mechanisms [8,9,10]. In contrast, evidence for secondary hyperalgesia outside the exposed field is more heterogeneous [6,12,16], where several studies report no or only subtle secondary hyperalgesia [2,4,5,7,9,11], while others demonstrated a clear extension of mechanical hypersensitivity beyond the irradiated region [6,8,10,12]. Furthermore, no UVA-induced secondary hyperalgesia could be observed by Harrison et al. or Hoffmann et al., though it should be noted that they did not observe any secondary hyperalgesia for UVB either [5,7]. These discrepancies likely arose from methodological differences rather than biological variability. Several experimental features appear to modulate the detectability of secondary phenomena. If the UVB intensity or irradiation area is too small, the temporal and spatial summation of nociceptive input may remain insufficient to engage central sensitization mechanisms [11]. Quantitative sensory testing methodology also plays a key role [6], as studies using low-intensity von Frey-filaments (~10 g) failed to identify secondary hyperalgesia [9], while those using higher-intensity von Frey filaments (~150 g) or pinprick stimulators (~25 g) were able to detect an extended area of sensitivity [6,8,10,17]. It is also suspected that the anatomical site influences detectability [16].

Importantly, when secondary hyperalgesia is observed, it is interpreted as evidence that UVB-induced inflammation can engage central nociceptive mechanisms beyond the irradiated area [5,8,9,10,11]. Persistent low-frequency nociceptive input from UVB-inflamed skin may trigger spinal sensitization, and descending facilitatory influences have been linked to the spread of mechanical hypersensitivity surrounding the site of inflammation [11]. Drummond et al. demonstrated that central nociceptive processing becomes altered during UVB-induced inflammation and can be additionally amplified through startle-evoked supraspinal activation [11]. In general, this model appears to primarily be a robust tool for inducing and studying primary inflammatory hyperalgesia, while secondary hyperalgesia can be induced in a reliable manner only under specific methodological conditions. From a methodological standpoint, these findings underscore that the detection of secondary hyperalgesia depends on a combination of factors, including the dose and size of the irradiated area [11], anatomical site [16], and mechanical testing parameters [6]. Therefore, it will be essential to carefully standardize and report these variables transparently to improve comparability between studies, and to maximize the potential of the UVB sunburn model for investigating both peripheral and central sensitization.

### 4.5. Towards a Standardization Framework

Building on the heterogeneity identified across the preceding domains, a structured standardization framework for the UVB sunburn model can be derived from the present synthesis. Future studies should, at a minimum, adhere to the following core parameters:

(1) Dose calibration should be MED-based and individually determined, with 2 MED recommended as the preferred dose to balance robust hyperalgesia induction against the risk of post-inflammatory hyperpigmentation.
(2) Irradiation source specifications, including the spectral range, peak wavelength, and irradiance, should be reported in full, as even nominally identical UVB sources may differ in erythemal efficacy.
(3) Irradiated areas should encompass at least 3 × 3 cm on a standardized anatomical site, preferably the volar forearm, to enable sufficient spatial summation for both primary and, where intended, secondary hyperalgesia assessment.
(4) Sensory testing should follow the standardized quantitative sensory testing battery, e.g., the German Research Network on Neuropathic Pain (DFNS) or an equivalent validated protocol.
(5) Temporal assessments should include serial measurements at baseline and at least at 24 and 48 h post-irradiation, as these intervals consistently captured peak hyperalgesia across the studies, with additional time points at 6, 72, and 96 h recommended for studies investigating full temporal profiles.

### 4.6. Limitations

This scoping review has several limitations that should be acknowledged. First, the number of included studies is relatively small (*n* = 12), which limits the generalizability of the synthesized findings and precludes formal quantitative comparison across methodological approaches. Second, considerable heterogeneity in the study designs, outcome measures, and reporting practices made direct comparison between studies difficult; in particular, several studies did not report all the relevant methodological details, such as irradiance values, skin phototype distribution or exact irradiated area dimensions, which constrained the depth of the present synthesis. Third, inherent to the scoping review design [14,15], no formal meta-analysis or quantitative risk-of-bias weighting was performed, and the narrative synthesis may therefore be subject to interpretive bias. Fourth, the restriction to published peer-reviewed studies may have introduced publication bias, as negative or inconclusive findings regarding, for instance, secondary hyperalgesia may be underrepresented in the literature. Finally, the proposed standardization recommendations in Section 4.5 are derived inductively from the reviewed evidence base rather than from a formal Delphi consensus process; therefore, these should be regarded as starting points for future methodological harmonization rather than as definitive guidelines.

## 5. Conclusions

In summary, this scoping review shows that UVB irradiation produces a well-defined inflammatory response accompanied by marked primary hyperalgesia, supporting the sunburn pain model as an approach for studying inflammatory pain in humans. Across the studies, erythema perfusion and primary hyperalgesia followed a delayed but consistent time course, with the interval between 24 and 72 h post-irradiation representing a relatively stable window for outcome assessment. In contrast, secondary hyperalgesia appeared more variably and was strongly influenced by methodological factors, indicating that central sensitization can only be reliably examined under optimized conditions. Although safety data remain limited, the available evidence indicates that using two MED supposably induces stable hyperalgesia while minimizing the risk of long-lasting post-inflammatory hyperpigmentation. By integrating the evidence on methodological approaches and response characteristics across the studies, the review identifies key sources of variability in the UVB model and offers a structured framework to support more consistent study design and interpretation in future experimental and pharmacological pain research.

## Figures and Tables

**Table 1:** Search strategies for different databases.

Database	Search Strategy
**PubMed**	(“sunburn”[Title/Abstract] OR “UVB”[Title/Abstract] OR “UV-B”[Title/Abstract] OR “ultraviolet B”[Title/Abstract] OR “UVR”[Title/Abstract] OR “ultraviolet radiation”[Title/Abstract] OR “UVB-induced”[Title/Abstract] OR “UV-induced”[Title/Abstract]) AND (“pain”[MeSH Terms] OR “pain”[Title/Abstract] OR “hyperalgesia”[Title/Abstract] OR “allodynia”[Title/Abstract] OR “nociception”[Title/Abstract] OR “sensitization”[Title/Abstract])
**Ovid**	(“sunburn” OR “UVB” OR “UV-B” OR “ultraviolet B” OR “UVR” OR “ultraviolet radiation” OR “UVB-induced” OR “UV-induced”) AND (“pain” OR “hyperalgesia” OR “allodynia” OR “nociception” OR “sensitization”) AND (“model” OR “models” OR “experimental” OR “study”)
**CINHAL**	(“sunburn” OR “UVB” OR “UV-B” OR “ultraviolet B” OR “UVR” OR “ultraviolet radiation” OR “UVB-induced” OR “UV-induced”) AND (“pain” OR “hyperalgesia” OR “allodynia” OR “nociception” OR “sensitization”) AND (“model” OR “models” OR “experimental” OR “study”)

**Table 2:** Characteristics of studies.

Author	Year	Country	*n*	Design	Sunburn Pain Model				Time Frame of Measurements	Research Question/Aim
					Wavelength	UV Dose	Localization	Size		
Hoffmann RT [7]	1999	Germany	10	Within-subject comparison	290–320 (UVB) 320–400 nm (UVA)	UVB: 1 × MED and 3 × MED UVA: 16,800 mJ/cm^2^ and 36,000 mJ/cm^2^	Upper leg (ventral side)	⌀ 1.5 cm	1, 6, 12, 24, 48, 96, 132 h	Time course and dose-dependency of erythema and hyperalgesia after UVA vs. UVB irradiation.
Benrath J [4]	2001	Germany	9	Dose–response study ± topical capsaicin pre-treatment	290–320 nm (UVB)	1–3 × MED (133, 266, 400 mJ/cm^2^) exposure times (15, 30, 45 s)	Volar forearm	⌀ 3 cm	3, 6, 9, 12, 24, 30, 36, 48, 60, 72, 96, 216 h	Time course and interrelation of skin blood flow and hyperalgesia; determination of neuropeptide-dependent mechanisms.
Siebenga PS [2]	2019	Netherlands	Study 1: 78 Study 2: 18	Study 1: longitudinal study on long-term effects after 3 × MED UVB; Study 2: within-subject comparison of UVB paradigm vs. control skin	290–320 nm (UVB)	Study 1: 3 × MED ≈251–1321 mJ/cm^2^ Study 2: 2 × MED ≈251–355 mJ/cm^2^	Study 1: upper back Study 2: upper back (right scapula)	Study 2: 3 × 3 cm	Study 1: >1751 days–750 days since irradiation Study 2: 1–36 h, 6 weeks, 6 months	Study 1: prevalence of post-inflammatory hyperpigmentation. Study 2: prevalence of post-inflammatory hyperpigmentation reliability of hyperalgesia.
Gustorff B [10]	2004	Austria	8	RCT crossover; two sessions, ≥7 days apart	290–320 nm (UVB)	3 × MED	Upper leg (ventromedial side)	⌀ 5 cm	20, 22, 24, 26, 28, 30 h	Stability and reproducibility of UVB-induced primary and secondary hyperalgesia.
Gustorff B [8]	2013	Austria	22	Two-part experimental study: (1) full QST at 24 h (*n* = 22); (2) time course of hyperalgesia 1–96 h (*n* = 12)	290–320 nm (UVB)	3 × MED	Upper leg (ventromedial side)	⌀ 5 cm	1, 2, 4, 8, 24, 32, 48, 72, 96 h	Magnitude and time course of primary and secondary hyperalgesia.
Harrison GI [5]	2004	United Kingdom	18	Within-subject comparison of SSR and UVA-I effects on opposite buttocks	340–400 nm (UVA-I) 280–400 nm (SSR: 92% UVB + 8% UVA)	Study 1: 1–3 × MED (SSR and UVA-I) Study 2: 3 × MED (SSR)	Buttock	Study 1: 2.5 × 2.5 cm Study 2: anulus with 6 cm diameter (2 cm central unirradiated zone)	3, 6, 9, 24, 48, 72 h	Dose- and time-dependent effects on thermal and mechanical pain sensitivity; determination of localization of mechanical sensitivity.
Bishop T [9]	2009	United Kingdom	Study 1: 12 Study 2: 12 Study 3: 12	Within-subject comparisons: Study 1: time and dose-dependence of UVB-induced sensory changes; Study 2: comparison of sensory changes induced by UVB (3 MED), 1% capsaicin, and thermal burn models; Study 3: distribution of changes in mechanical sensitivity around annular UVB lesion and annular 1% capsaicin lesion	290–320 nm (UVB)	Study 1: 1–3 × MED~476 ± 20.6 mJ/cm^2^ Study 2: 3 × MED~439 ± 28.3 mJ/cm^2^ Study 3: 3 × MED~454 ± 25.7 mJ/cm^2^	Volar forearm	Study 1: 1 × 1 cm Study 2: 3.2 × 3.2 cm Study 3: anulus with 5 cm diameter (2 cm central unirradiated zone)	2, 4, 6, 24, 48, 72, 96 h	Characterization of time course and dose-dependence of UVB-induced inflammation and sensory changes; comparison with thermal burn and capsaicin pain models.
Mørch CD [16]	2013	Denmark	15	Within-subject comparison with test–retest reliability	290–320 nm (UVB)	3 × MED ≈55–160 mJ/cm^2^	Ventro-medial side of the upper arm	⌀ 1.5 cm	Baseline, 24, 48, 72 h	Test–retest reliability and estimates of the required sample size for pharmacological screening.
Lo Vecchio S [6]	2014	Denmark	24	Two-part experimental study: (1) UVB experiment (*n* = 16): standardized UVB-induced inflammation on forearm and lower back; (2) EMLA experiment (*n* = 8): same UVB procedure + topical lidocaine/prilocaine cream (EMLA) on irradiated and control arm	290–320 nm (UVB)	3 × MED	Middle of the forearm and lower back	3 × 4 cm	Baseline, 24 h	Changes in cutaneous blood flow and mechanical pain sensitivity; evaluation of the effect of topical anesthesia.
Lo Vecchio S [12]	2015	Denmark	16	Within-subject comparison combining UVB-induced cutaneous inflammation with exercise-induced deep tissue sensitization	290–320 nm (UVB)	3 × MED	Upper trapezius and ipsilateral lower back	3 × 4 cm	Baseline, 24 h	Interactions between UVB-induced cutaneous and DOMS-induced deep tissue hyperalgesia regarding blood flow; pinprick- and mechanically induced hyperalgesia; and temporal summation.
Lötsch J [13]	2018	Germany	82	Two-group design; UVB vs. capsaicin,	290–320 nm (UVB)	2 × MED	Volar forearm	1 cm^2^	Baseline, 24 h	QST changes induced by UVB and capsaicin hypersensitization.
Drummond PD [11]	2025	Australia	31	two experimental sessions (24 h apart)	290–320 nm (UVB)	~0.39–1.5 mW/cm^2^ exposure time (65–250 s)	Volar forearm	⌀ 1 cm	Baseline, 24 h	Effects on supraspinal nociceptive processing.

## Data Availability

No new data was generated for this review.

## Supplementary Materials

The following supporting information can be downloaded at: https://www.mdpi.com/article/10.3390/life16040662/s1, Table S1. PRISMA 2020 checklist. Reference [18] is cited in the Supplementary Materials.
